# Rooks and Retribution

**Published:** 1891-08-22

**Authors:** 


### PASSING TOPICS.

### ROOKS AND RETRIBUTION.

These are late days for insisting upon the rudimentary proposition that the economy of nature is not to be roughly disturbed unless we are prepared for certain more or less disastrous consequences. One would have supposed that ample opportunities had been afforded to all students of agriculture to master a lesson which has been taught through long ages, for is not agriculture the very oldest of the arts, and rightly described as being the foundation of all other arts and coeval in all countries with the first dawn of civilization?

Nevertheless there comes from our Northern counties a tale of distress which seems to be the obvious and unavoidable sequel to a grievous breach of elementary rules—the wholesale destruction of insectivorous birds resolved upon by the Northumberland farmers earlier in the year. "The rooks," writes a correspondent graphically, "have been cleared out," and as a natural consequence the caterpillars are having it all their own way in the turnip field and elsewhere. If any grain of satisfaction is to be gleaned, it is to be found in the reflection that Nemesis has overtaken the offenders, though there is too much reason to fear that many innocent people are likely to suffer too, for caterpillars are not gifted, as far as we know, with a nice sense of discrimination.

Alas! that anybody should put himself in fault in respect of the very matters wherein the world is only too ready to accept him as an expert and to follow his example. A man may be pardoned, and possibly he may be commended, for ignorance of his neighbour's business, but what are we to say of him, when he proves incapable of understanding his own? Our farmers have too many difficulties already to contend with to permit of their adding to the number with safety. No wonder the Royal Agricultural Society is bestirring itself, and that the papers—not only those published in the farming interest, but the daily journals to boot—teem with articles and correspondence upon things bucolic. Perhaps the most curious statement of all is that this extirpation of the farmers' best friends and helpers has been ordered in the name of Science! Until corroboration is forthcoming we will not believe that the warrant issued with her signature is anything but a forgery. We decline absolutely to accept the assertion, that science is capable of decreeing

"A slaughter told in groans, not words,
The very St. Bartholomew (no reflection on the Hospital) of birds."

If our agricultural colleges did turn out to be the disseminators of such teaching, we should recommend practical farmers to give up reading the treatises issued under their high sanction, and to take to their Longfellow. Copies of a cheap reprint of "The Birds of Killingworth," and other similar productions, might be distributed in agricultural districts with advantage, and not in the interests of the rooks and their fellows only, but of the farmers, the public, and common sense. Agricola.

